# Supplementary material for: Form-Stable Composite Phase Change Materials Based on Porous Copper–Graphene Heterostructures for Solar Thermal Energy Conversion and Storage
Source: Polymers (Basel). 2023 Dec 16;15(24):4723. doi: 10.3390/polym15244723 (PMC10748072; doi:10.3390/polym15244723)
Supplement: Supplementary file 1 [file polymers-15-04723-s001.zip › polymers-2728198-supplementary.pdf]

Supplementary Information

Form-Stable Composite Phase Change Materials Based on Porous Copper–Graphene Heterostructures for Solar Thermal Energy Conversion and Storage

Chao Chang *, Bo Li, Baocai Fu, Xu Yang and Yulong Ji

Institute of Marine Engineering and Thermal Science, Marine Engineering College, Dalian Maritime University, Dalian 116026, China

* Correspondence: chaochang@dlmu.edu.cn

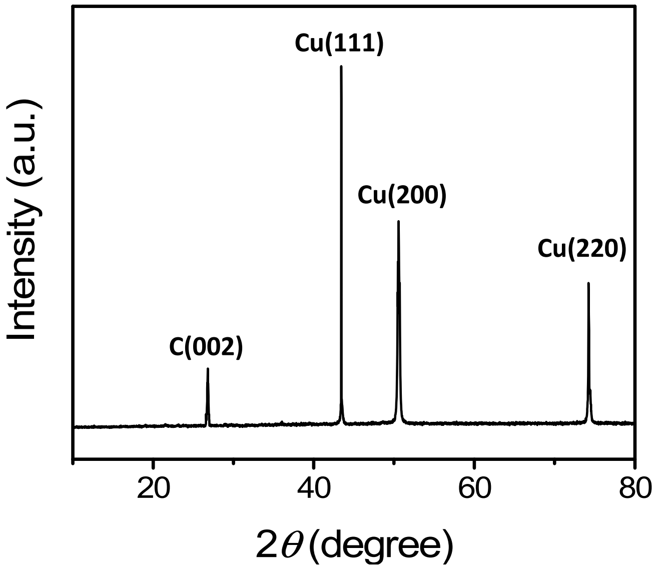

Figure S1. XRD patterns o of the G–Cu heterostructures.

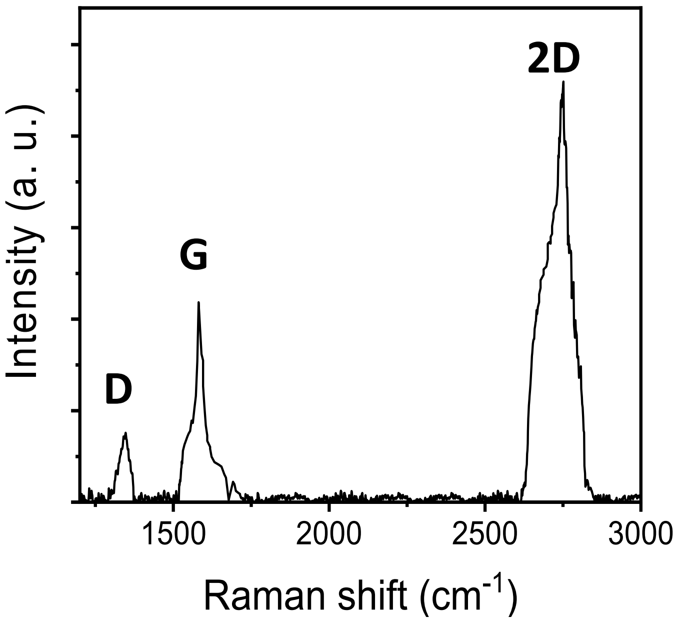

Figure S2. Raman spectrum from the out surface of the G–Cu heterostructures.

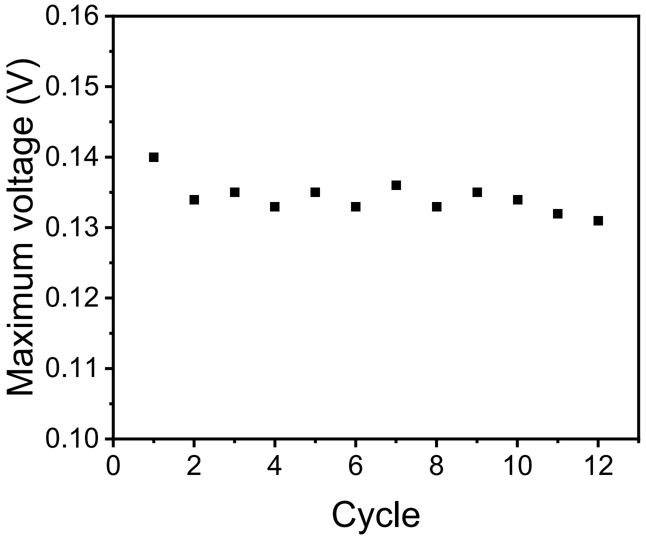

Figure S3. Stability tests of the solar-electric conversion device.
